# Association of BrERF72 with methyl jasmonate-induced leaf senescence of Chinese flowering cabbage through activating JA biosynthesis-related genes

**DOI:** 10.1038/s41438-018-0028-z

**Published:** 2018-05-01

**Authors:** Xiao-li Tan, Zhong-qi Fan, Wei Shan, Xue-ren Yin, Jian-fei Kuang, Wang-jin Lu, Jian-ye Chen

**Affiliations:** 1 State Key Laboratory for Conservation and Utilization of Subtropical Agro-bioresources/Guangdong Provincial Key Laboratory of Postharvest Science of Fruits and Vegetables/Guangdong Vegetables Engineering Research Center, College of Horticulture, South China Agricultural University, 510642 Guangzhou, China; 2 The State Agriculture Ministry Laboratory of Horticultural Plant Growth, Development and Quality Improvement, College of Agriculture and Biotechnology, Zhejiang University, Zijingang Campus, 310058 Hangzhou, China

## Abstract

The ethylene response factor (ERF) and phytohormone jasmonate (JA) are reported to function in leaf senescence. The involvement of ERF in JA-mediated leaf senescence, however, needs to be elucidated. In the present work, we demonstrate a Chinese flowering cabbage ERF transcription factor (TF), BrERF72, that is associated with JA-promoted leaf senescence. Exogenous application of methyl jasmonate (MeJA)-accelerated leaf senescence of Chinese flowering cabbage, evidenced by the data that MeJA treatment led to the stronger reduction in the maximum quantum yield (Fv/Fm), photosynthetic electron transport rate (ETR), and total chlorophyll content, while significant induction in the expression of several senescence-associated genes (*SAGs*) including *BrSAG12, BrSAG19*, and chlorophyll catabolic genes (*CCGs*) *BrPAO1*, *BrNYC1*, *BrPPH1*, and *BrSGR1*. Increases in levels of endogenous JA and transcripts of JA biosynthetic genes *BrLOX4*, *BrAOC3*, and *BrOPR3* were also found after MeJA treatment. BrERF72 was a MeJA-inducible, nucleus-localized protein, and possessed trans-activation ability. Transient overexpression of *BrERF72* in tobacco leaves also promoted leaf senescence. More importantly, further experiments revealed that BrERF72 directly activated expression of *BrLOX4*, *BrAOC3*, and *BrOPR3* through binding to their promoters via the GCC or DRE/CRT *cis*-element. Together, the novel JA-ERF association reported in our study uncovers a new insight into the transcriptional regulation of JA production mediated by ERF during JA-promoted leaf senescence in Chinese flowering cabbage.

## Crop genetics: the secrets of aging in leaves

Researchers in China have uncovered a gene involved leaf aging in response to the plant hormone jasmonate in Chinese flowering cabbage. A team led by Jian-ye Chen of the South China Agricultural University treated Chinese flowering cabbage with jasmonate, which is known to promote leaf senescence in other species. In addition to accelerating senescence, the treatment caused the cabbage to produce more jasmonate. The team also used RNA sequencing to discover a new transcription factor, *BrERF72*, linking jasmonate and leaf senescence. *BrERF72* is active during leaf senescence and was more strongly expressed in jasmonate-treated leaves. The team found that *BrERF72* activates jasmonate biosynthesis genes, potentially creating a feedback loop reinforcing senescence. These findings extend our understanding of how leaves age, which will help improve breeding, treatment, and storage of leafy crops.

## Introduction

As the final phase of development, leaf senescence is considered as an important biological process in plants that nutrition substances in senescing leaves were reallocated to the newly formed organs, which can maximize plants viability in the next year^1–3^. However, precocious or abnormal senescence induced by stresses or other factors often shorten the growth stage of crops, resulting in a loss in crop yield and products quality^4,5^. In addition, leaf senescence limits the shelf-life and nutritional value of many leafy vegetables, representing the main postharvest problem of these vegetables^6,7^. Therefore, in agricultural aspects, understanding the regulatory mechanisms of leaf senescence is critical for the breeding of higher-yielding and well-nutrition crops, as well as for the better management to prevent postharvest loss.

The initiation and development of leaf senescence is tightly controlled by various endogenous and environmental factors, such as developmental stage, phytohormones, nutrients, and stresses^2,8,9^. Jasmonates (JAs), a group of oxylipin compounds ubiquitous in plants, act as a crucial signal to modulate diverse physiological processes including leaf senescence^10^. In Arabidopsis, the endogenous JA level increases dramatically during leaf senescence^11^. Moreover, exogenously applied JA or its bioactive derivatives methyl jasmonate (MeJA) stimulate leaf senescence in several plants such as Arabidopsis^11–14^, wormwood^15^, barley^16^, maize^17^, and rice^18^, by upregulating some senescence-associated genes (*SAGs*). Thus, JA is an important factor in inducing leaf senescence ^10,19^.

During the past decades, great achievements have been made in JA biosynthesis, perception, and signaling in higher plants^20–22^. Briefly, JA and its derivatives are biosynthetized from α-linolenic acid (18:3) (α-LeA), then α-LeA is converted to 12-oxo-phytodienoic acid (12-OPDA) in several reactions catalyzed by 13-lipoxygenase (LOX), allene oxide synthase (AOS), and allene oxide cyclase (AOC), respectively. 12-OPDA is subsequently converted to JA by the involvement of OPDA reductase3 (OPR3) following three cycles of ß-oxidation^20,22^. The JA signal is perceived by its receptor CORONATINE INSENSITIVE1 (COI1) to form a stable COI1/JA complex. This complex then interacts with JA ZIM-domain (JAZ) repressors to degrade JAZ through the 26S proteasome pathway, and to liberate various downstream transcription factors (TFs)^20–22^. These TFs, including bHLH^14,23,24^, MYB^25^, WD-bHLH-MYB transcription complex^26^, and WRKY^13^, are essential for JA-mediated physiological processes. Interestingly, a TEOSINTE BRANCHED/CYCLOIDEA/PCF (TCP) TF TCP4 directly activates the expression of JA biosynthetic gene *LOX2*, while the repression of TCP4 by miR319 suppresses JA biosynthesis and delays leaf senescence in Arabidopsis^27^. Another TCP TF TCP20 inhibits *LOX2* and the *tcp20* mutant exhibits an earlier onset of leaf senescence^28^. These data demonstrate that activation of JA biosynthesis induces senescence-related genes expression and promotes leaf senescence. Although JA biosynthesis and signaling networks have been intensively investigated, as well as leaf senescence promoted by JA, the upstream TFs involved in leaf senescence by controlling its level remain to be explored.

Despite large TF families such as NO APICAL MERISTEM/ARABIDOPSIS TRANSLATION ACTIVATIONFACTOR/CUP-SHAPED COTYLEDON (NAC), WRKY, bHLH, APETALA2/ETHYLENE RESPONSE FACTOR (AP2/ERF), and MYB are involved in leaf senescence, consisting of a hierarchical and coordinated regulatory network^22,29,30^, only several TFs, including WRKY53^31^, WRKY54 and WRKY70^32^, WRKY57^13^, ANAC092/ANAC019/055/072^19^, bHLH03/bHLH13/bHLH14/bHLH17^14^, and MYC2/MYC3/MYC4^19^, are identified to be positively or negatively involved in JA-promoted leaf senescence. Many studies have demonstrated that AP2/ERF TFs not only act as important regulators of ethylene signaling^33^, but also are involved in many physiological processes controlled by JA^34^. Yet, little is known about the regulatory mechanisms of AP2/ERFs associated with JA-promoted leaf senescence.

Leafy vegetables like Chinese flowering cabbage/choy sum are rich in nutraceutical and health-promoting metabolites^35^. But leafy vegetables senesce rapidly after harvest, resulting in substantial loss and short shelf-life due to yellow leaves. Our previous study showed that a leaf senescence-induced TF BrWRKY65 directly activated three *SAGs* in Chinese flowering cabbage^7^. Nevertheless, the underlying transcriptional regulation of Chinese flowering cabbage leaf senescence is still largely unclear. Here, we reported that MeJA pretreatment accelerated leaf senescence of Chinese flowering cabbage. More importantly, we further identified a novel transcriptional activator AP2/ERF TF BrERF72 that associated with leaf senescence by directly regulating JA biosynthetic genes *BrLOX4*, *BrAOC3*, and *BrOPR3*. Our findings thus provide novel evidence about the transcriptional regulation of JA-mediated leaf senescence involving ERF TFs.

## Materials and methods

### Plant materials and treatments

Chinese flowering cabbages (*Brassica rapa* var. parachinensis) were harvested after ~40 days of growth. These harvested cabbages were pre-cooled and transported to the lab immediately. Uniform and no damaged cabbages were chosen and randomly divided into two groups for the following treatments.

For MeJA treatment, the cabbages were sprayed with 100 µM MeJA (containing 0.2% Tween-20). The cabbages sprayed with distilled water were served as control. Both control and MeJA-treated cabbages were subsequently put into plastic baskets (20 per box), which were packed with polyethylene-perforated plastic bags (0.03 mm thickness, with six holes of 1 cm in diameter at each side), and stored in the incubators at 15 °C. Immediately after MeJA treatment and after 1, 3, 5, and 7 days of storage, the second leaf from the bottom of ten cabbages were collected for physiological and molecular analysis. The collected leaves were rinsed three times with distilled water containing 0.2% Tween-20 to remove the residual MeJA on the leaf surface, frozen in liquid nitrogen, and stored at –80 °C for further use.

### Leaf senescence evaluation

Senescence-associated physiological markers, such as total chlorophyll content and chlorophyll fluorescence, such as the maximal PS II quantum yield (Fv/Fm) and photosynthetic electron transport rate (ETR), were measured to evaluate leaf senescence. Leaf tissues (~2.5 g) were ground to power using liquid nitrogen, and total chlorophyll was extracted with 80% cold acetone overnight at 4 °C, and then estimated spectrophotometrically at 663 and 645 nm^36^. Fv/Fm and ETR were calculated non-invasively using an Imaging-PAM-M series chlorophyll fluorometer (Heinz Walz, Effeltrich, Germany) integrated with a CCD camera that enables to capture digital images of the emitted fluorescence with high resolution^37^.

### Quantification of endogenous JA content by enzyme-linked immunosorbent assay (ELISA)

About 1.0 g of cabbage leaf was used for the analysis. The extraction of JA and measurement of ELISA using monoclonal antibodies of JA were performed as described in previous studies^38^.

### RNA extraction, cDNA synthesis, gene cloning, and sequence analysis

Total RNA of sampled leaves was isolated using the RNeasy Plant Mini Kit (Qiagen, Germany). cDNA used as the template for reverse transcription polymerase chain reaction (RT-PCR) and quantitative real-time PCR (qRT-PCR) was synthesized from 1 µg of total RNA using a PrimeScript^TM^ RT reagent kit (Takara, Japan). According to Chinese cabbage *chiifu* genome in BRAD (http://brassicadb.org/brad/), the coding region of BrERF72 was cloned through RT-PCR with gene-specific primers. PCR products were sequenced and blasted in the NCBI database for the homology identification. Basic information of BrERF72 protein such as theoretical isoelectric point (pI) and mass value was calculated using the online database (http://web.expasy.org/compute_pi/). CLUSTALW (version 1.83), GeneDoc, and MEGA5 software were used for the alignment and phylogenetic tree of AP2/ERF proteins, respectively.

### Analysis of gene expression with qRT-PCR

qRT-PCR was carried out using GoTaq® qPCR Master Mix Kit (Promega, USA) in a Bio-Rad CFX96 Real-Time PCR System (Bio-Rad, USA). *EF-1-α* (GO479260) was adopted as the reference gene to normalize the transcript levels of each gene, using the cycle threshold (*C*t) value.

### Subcellular localization analysis

The full length of *BrERF72* without stop codon was subcloned into the pEAQ-GFP vector^39^ to fuse with the green fluorescent protein (GFP) gene sequence. Then, the pEAQ-BrERF72-GFP or the control pEAQ-GFP vector was transferred into the *Agrobacterium tumefaciens* strain GV3101 by electroporation, and transiently expressed in tobacco (*Nicotiana benthamiana*) leaves as described previously^40^. After 48 h of infiltration, infected leaf tissues were collected for analysis. GFP signal was captured using a fluorescence microscope (Zeiss Axioskop 2 Plus).

### Transcriptional activation analysis in yeast cells

First, *BrERF72* was inserted into the pGBKT7 vector (Clontech, USA) to fuse with GAL4 DNA-binding domain. Then, the fusion construct pGBKT7-BrERF72, positive control (pGBKT7-53 + pGADT7-T), and negative control (pGBKT7 vector) were transformed into yeast cells Gold Y2H using the lithium acetate method. The transcriptional activation ability of BrERF72 was evaluated based on the growth status and α-galactosidase activity of yeast cells that grow on SD medium without tryptophan (SD/-Trp), or tryptophan, histidine, and adenine (SD/-Trp-His-Ade).

### Promoter analysis

DNeasy Plant Mini Kit (Qiagen, Germany) was used to isolate genomic DNA of cabbage leaves. The promoters of three JA biosynthetic genes *BrLOX4*, *BrAOC3*, and *BrOPR3* were amplified with nested PCR using a Genome Walker Kit (Clontech). Conserved *cis*-element motifs presented in each promoter was predicted in Plant-CARE database (http://bioinformatics.psb.ugent.be/webtools/plantcare/html/).

### Electrophoretic mobility shift assay (EMSA)

The coding sequence of BrERF72 was subcloned into the pGEX-4T-1 (Amersham Biosciences) vector to produce recombinant GST-BrERF72 protein in *Escherichia coli* strain BM Rosetta (DE3). The induced GST-BrERF72 protein was further purified using glutathione-superflow resin (Clontech) according to the manufacturer’s protocol. The 5′ ends of synthesized oligonucleotide probes were labeled with biotin. EMSA was performed using the LightShift Chemiluminescent EMSA Kit (Thermo Scientific) as previously described^7,40^. Briefly, GST-BrERF72 protein and biotin-labeled probes were incubated together, then free and protein–DNA complexes were separated by 6% native polyacrylamide gel electrophoresis, transferred onto nylon membrane and detected by a ChemiDoc™ MP Imaging System (Bio-Rad, USA) using the chemiluminescence method. Unlabeled and mutated probes, as well as GST protein alone, were used as competitors and negative control, respectively.

### Dual-luciferase reporter assays in tobacco leaves

For transcriptional activity analysis of BrERF72, its coding sequence was cloned into the 35S promoter-drove pBD vector to fuse with the yeast GAL4 DNA-binding domain (GAL4BD) as effector (pBD-BrERF72). The double-reporter vector contains a firefly luciferase (LUC) driven by five copies of the GAL4-binding element (5 × GAL4) and minimal TATA region of CaMV 35S. Renilla luciferase (REN) in the same vector drove by the 35S promoter was used for normalization. For assaying the binding activity of BrERF72 to the promoters of *BrLOX4*, *BrAOC3*, and *BrOPR3*, their promoters were inserted into pGreenII 0800-LUC double-reporter plasmid as reporters^41^. Effector plasmid was constructed by inserting BrERF72 into the pEAQ vector. The constructed effectors and reporters plasmids with different combinations were co-transformed into tobacco leaves mentioned above.

Activities of LUC and REN luciferase were quantified after 48 h of infiltration using the dual luciferase assay kit (Promega, USA) on the Luminoskan Ascent Microplate Luminometer (Thermo Fisher Scientific, USA). The transcriptional ability of BrERF72 was assessed by the LUC to REN ratio. Six biological repeats were included for each pair.

### Transient overexpression of BrERF72 in tobacco leaves

Transient overexpression of pEAQ-BrERF72 mentioned above were performed in tobacco (*Nicotiana tabacum*) leaves as described previously^42,43^. After infiltration, tobacco plants were maintained in the chamber and the infiltrated leaves were sampled for photos.

### Data analysis

Data were recorded as mean ± standard errors (SE) of three or six independent biological replicates. Statistical differences between samples (*P* < 0.05 or 0.01) were determined by Student’s *t* test.

### Primers

All primers designed and used in this work are presented in Supplementary Table S1.

## Results

### Exogenous application of MeJA promotes leaf senescence of Chinese flowering cabbage

**Fig. 1:**
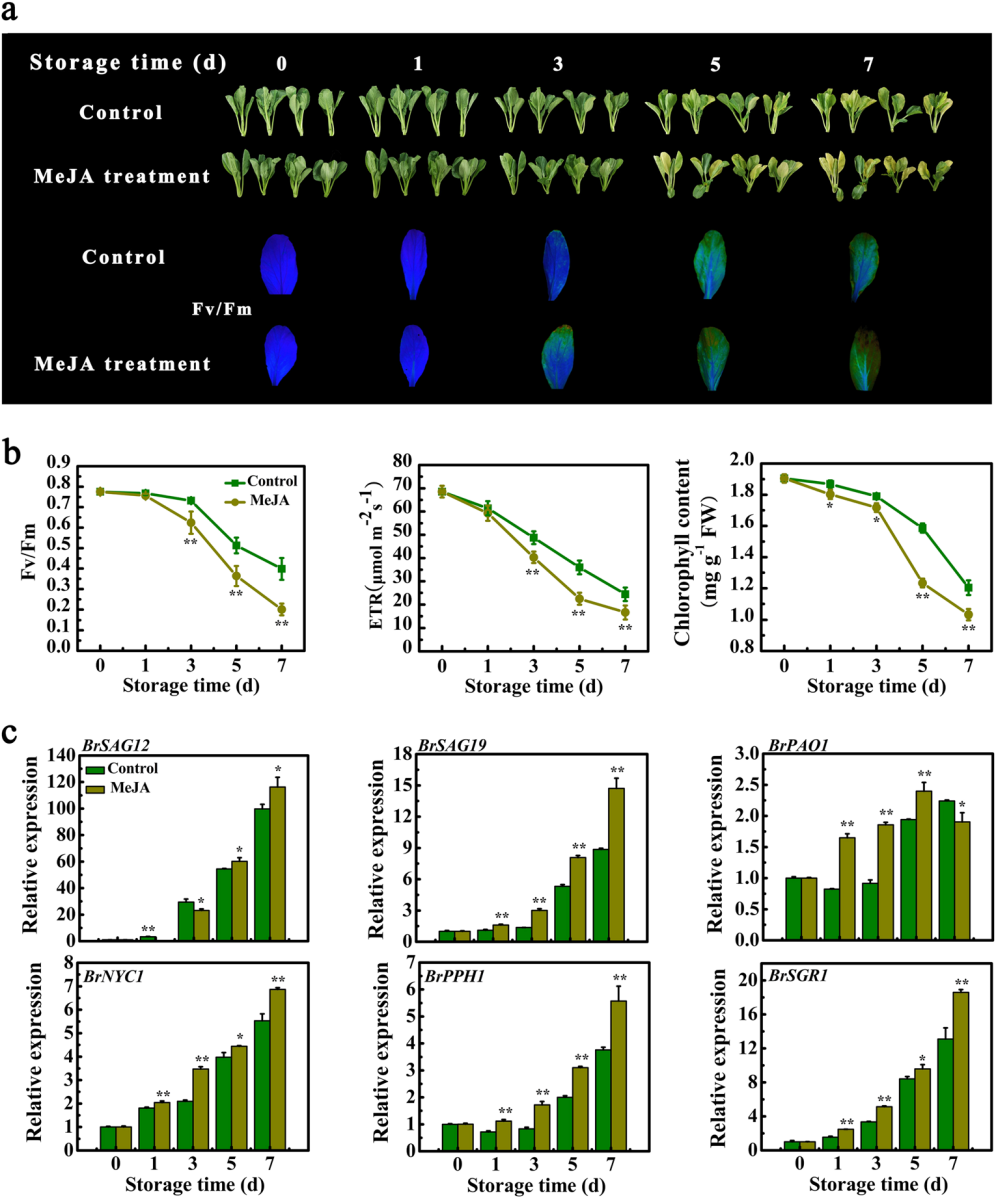
Application of MeJA promotes leaf senescence of Chinese flowering cabbage. **a** Appearance and chlorophyll fluorescence imaging with Fv/Fm of control and MeJA-treated cabbage leaves during senescence. **b** Changes of Fv/Fm, ETR, and chlorophyll content in control and MeJA-treated cabbage leaves during senescence. **c** Relative expression of six *SAGs* including *BrSAG12*, *BrSAG19*, *BrPAO1*, *BrNYC1*, *BrPPH1*, and *BrSGR1* in control and MeJA-treated cabbage leaves during senescence. Data presented in **b** and **c** are the mean ± SE of three biological replicates. Asterisks indicate a significant difference in MeJA-treated leaves compared with control leaves (Student’s *t* test: *P < 0.05 and **P < 0.01)

The leaves of control and MeJA-treated cabbages started to turn yellow after 5 and 3 days of storage, respectively, and became serious thereafter. Furthermore, the leaves of MeJA-treated cabbages showed more obvious yellowing than control leaves during senescence (Fig. 1a). Harvested green leaves still have the ability of photosynthesis, which is affected by senescence. To monitor spatial and temporal changes in photosynthetic parameters in leaves, noninvasive chlorophyll fluorescence imaging was used. In agreement with more yellow areas developed in MeJA-treated leaves, the maximum quantum yield (Fv/Fm) and photosynthetic electron transport rate (ETR) reduced faster and stronger in the MeJA-treated leaves than those of the control (Fig. 1a, b). Accordingly, total chlorophyll content was obviously lower in MeJA-treated leaves (Fig. 1b). We also compared the expression of *SAGs* including *BrSAG12*, *BrSAG19*, and chlorophyll catabolic genes (*CCGs*) such as *BrPAO1*, *BrNYC1*, *BrPPH1*, and *BrSGR1* encoding pheophorbide a oxygenase (PAO), NON-YELLOW COLORING (NYC), pheophytin pheophorbide hydrolase (PPH), and STAY-GREEN (SGR), respectively, between MeJA-treated and control leaves. As shown in Fig. 1c, transcript levels of all *SAGs* were obviously increased during leaf senescence. Moreover, MeJA treatment obviously increased the transcripts of those genes compared to control, with a 1.18-, 1.67-, 1.29-, 1.48-, and 1.41-fold induction for *BrSAG12*, *BrSAG19*, *BrNYC1*, *BrPPH1*, and *BrSGR1* at 7 days, and a 1.21-fold induction for *BrPAO1* at 5 days of storage, respectively (Fig. 1c). Therefore, all of the results above imply that exogenous application of MeJA promotes leaf senescence of Chinese flowering cabbage.

### Exogenous MeJA increases endogenous level of JA and enhances expression of JA biosynthetic genes *BrLOX4*, *BrAOC3*, and *BrOPR3*

**Fig. 2:**
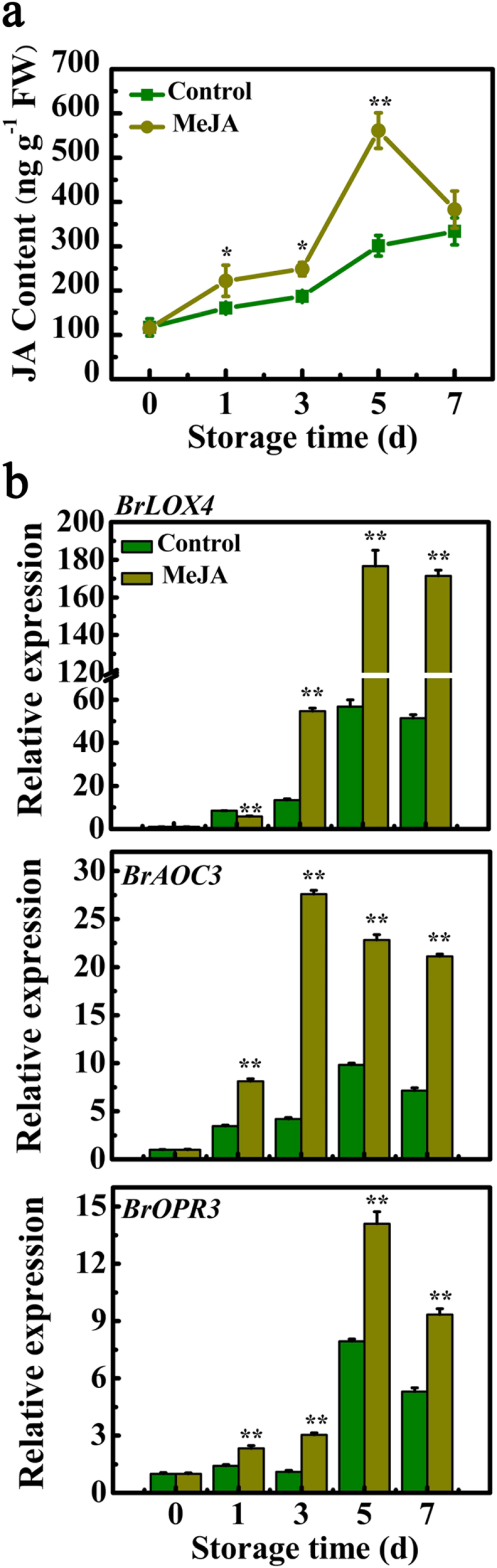
Application of MeJA increases endogenous JA level and enhances expression of JA biosynthetic genes during leaf senescence of Chinese flowering cabbage. **a** Changes of endogenous JA level. **b** Expression of JA biosynthetic genes including *BrLOX4*, *BrAOC3*, and *BrOPR3*. Each value represents the mean ± S.E. of three biological replicates. Asterisks indicate a significant difference between control and MeJA treatment by student’s *t* test: *P < 0.05 and **P< 0.01

To test whether endogenous JA level was changed by exogenous application of MeJA, we measured the JA concentration in cabbage leaves during senescence. The endogenous JA in control or MeJA-treated leaves accumulated during senescence, and MeJA-treated leaves had significantly higher level than the control (Fig. 2a). At 5 days of storage, the JA level in leaves of MeJA treatment was 561.19 ng/mg FW, which was about 0.86-fold higher than that of the control leaves (301.24 ng/mg FW) (Fig. 2a). We further speculated that JA biosynthetic gene expressions were influenced by MeJA treatment. As expected, qRT-PCR analysis showed that consistent with the change of JA content, transcription levels of JA biosynthetic genes, including *BrLOX4, BrAOC3*, *and BrOPR3*, were significantly upregulated in MeJA-treated leaves (Fig. 2b). Thus, it appears that exogenous MeJA increases endogenous concentration of JA during leaf senescence, possibly through induction of JA biosynthetic genes.

### Identification and molecular characterization of BrERF72

**Fig. 3:**
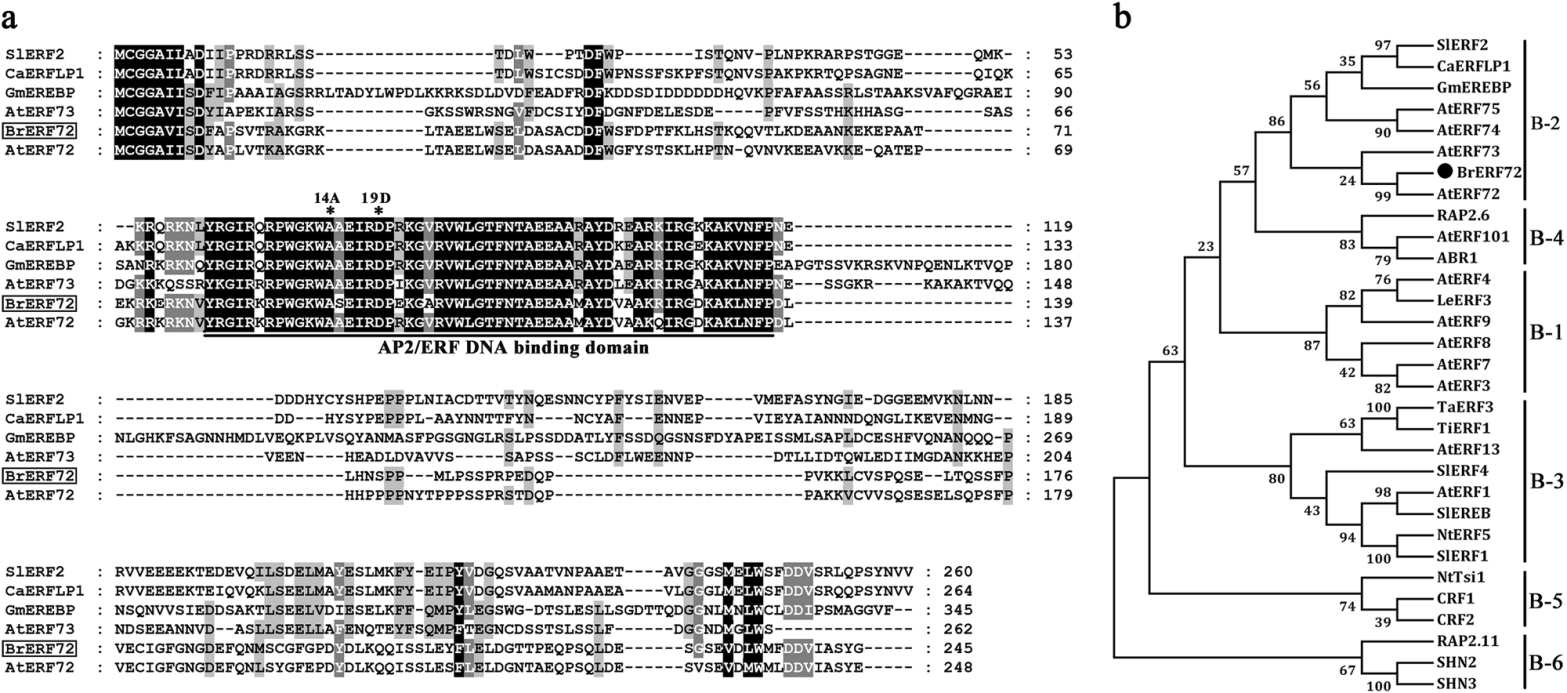
Sequence and phylogenetic analyses of BrERF72. **a** Multiple alignment of the deduced BrERF72 protein with other plant ERF proteins including tomato SlERF2, pepper CaERFLP1, soybean GmEREBP, Arabidopsis AtERF72, and AtERF73. Identical and similar amino acids were shaded in black and gray, respectively. The conserved AP2 domain is underlined. The conserved amino acid residues of 14th adenine (A) and 19th aspartate (D) in the AP2 domain are marked by asterisks. **b** Phylogenetic analysis of BrERF72 and ERFs from different species. BrERF72, along with Arabidopsis AtERF72 and AtERF73, and tomato SlERF2 were classified into B-2 subgroup of ERFs. Multiple alignment was carried using CLUSTALW and the phylogenetic tree was constructed with MEGA6.0 using a bootstrap test of phylogeny with neighbor-joining test and default parameters

According to our RNA-seq and Chinese cabbage genome (http://brassicadb.org/brad/) databases, notably, a putative *AP2/ERF* gene (XM_009137250.2) was notified to be obviously induced by leaf senescence. Homology search revealed that its sequence shared the highest identity with Arabidopsis thaliana AtERF72 (79%), thus it was termed as *BrERF72*. The length of open reading frame (ORF) of BrERF72 is 738 bp, which encodes a protein of 245 amino acids. Its predicted molecular weight and isoelectric point (pI) of BrERF72 are 27.42 kDa and 4.94, respectively. Sequence alignment showed that BrERF72 contains one highly conserved AP2 DNA-binding domain, containing ~60 amino acids from 80 to 137 aa (Fig. 3a). The conserved 14th adenine (A) and 19th aspartate (D), which are vital for DNA-binding sequences recognition^44^, were also found in BrERF72 (Fig. 3a). To know the classification of BrERF72, a phylogenetic tree was constructed, showing that BrERF72 is closely related to AtERF72, and both are members of ERF subfamily group B-2 (Fig. 3b).

**Fig. 4:**
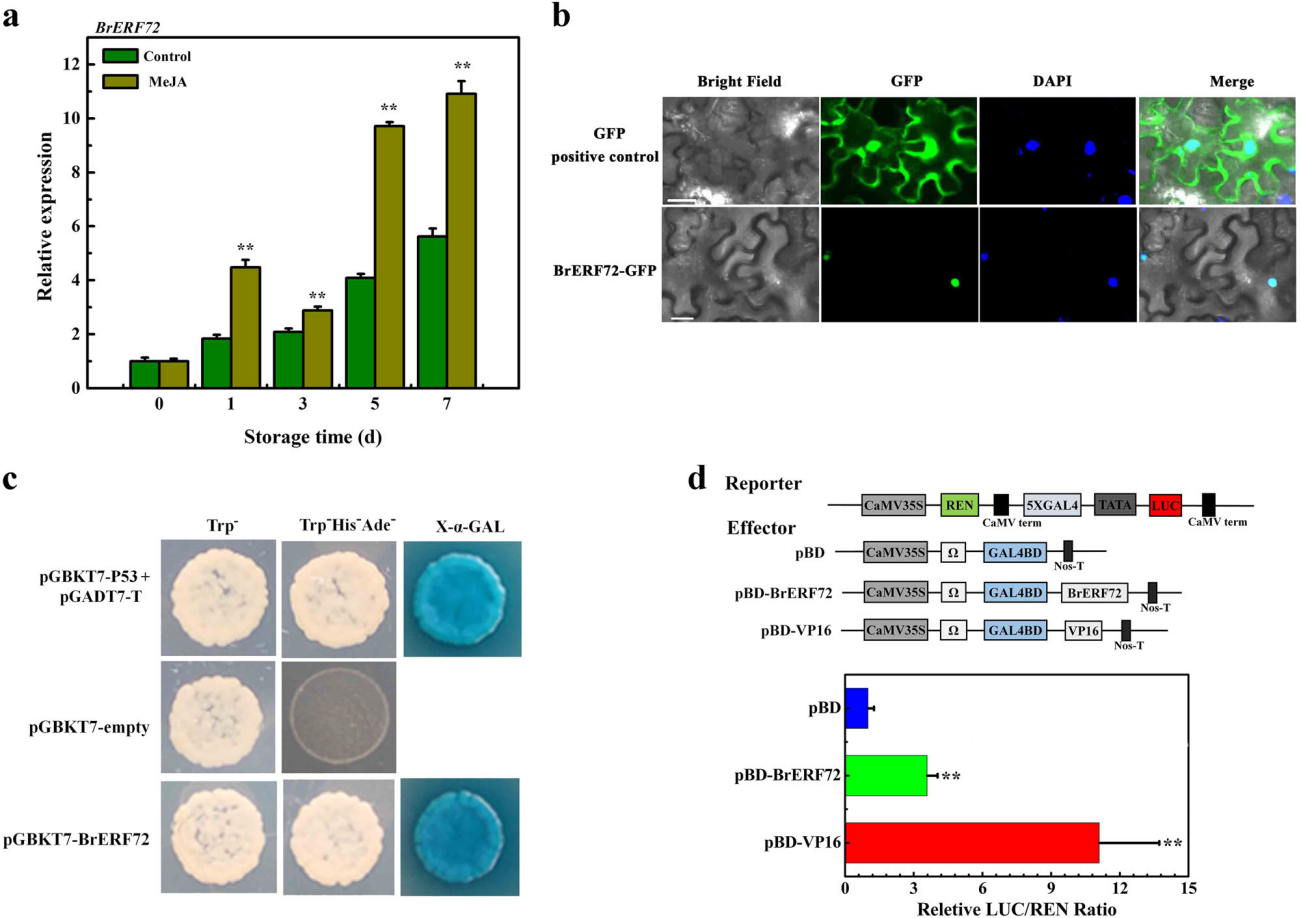
Molecular characterization of BrERF72. **a** Relative expression of *BrERF72* in control and MeJA-treated cabbage leaves during senescence. Each value represents the mean ± SE of three biological replicates. Asterisks indicate a significant difference in MeJA-treated leaves compared with control leaves (Student’s *t* test: *P < 0.05 and **P < 0.01). **b** Subcellular localization of BrERF72 in tobacco leaves. The fusion protein (BrERF72-GFP) and GFP-positive control were transiently expressed in *Nicotiana benthamiana* leaves, respectively, by *Agrobacterium tumefaciens* strain GV3101. GFP fluorescence was observed with a fluorescence microscope. GFP fluorescence was observed with a fluorescence microscope. The 4,6-diamidino-2-phenylindole (DAPI) staining indicates the localization of nuclei. Merged images show colocalization of GFP and DAPI signals. Bars, 25 μm. **c** Transcriptional activation of BrERF72 in yeast cells. The coding region of BrERF72 was cloned into the pGBKT7 (GAL4DBD) vector to construct the DBD-BrERF72 construct. DBD-BrERF72, together with the positive control (p53 + T-antigen) and negative control (pGBKT7) were transformed into yeast cells. Yeast clones transformed with different constructs were grown on SD plates without tryptophan or without tryptophan, histidine, and adenine for 3 days at 30 °C. Transcription activation was monitored according to growth status of yeast cells and an α-Gal assay. **d** Transcriptional activation activity of BrERF72 in tobacco leaves. The trans-activation ability of BrERF72 is indicated by the ratio of LUC to REN. The LUC/REN ratio of the empty pBD vector (negative control) was used as a calibrator (set as 1). pBD-VP16 was used as a positive control. Each value represents the mean ± SE of six biological replicates. The asterisk indicates a significant difference at the 1% level compared to the pBD

Similar with the RNA-seq data, qRT-PCR analysis showed that expression of *BrERF72* was induced during leaf senescence. Moreover, more remarkably increase of *BrERF72* transcript was observed in MeJA-treated leaves, with ~0.9- and 0.5-fold higher than that of the control leaves at 5 and 7 days of treatment, respectively (Fig. 4a). To know the subcellular localization of BrERF72, a BrERF72 and green fluorescent protein (GFP) fusion construct was prepared, and transiently expressed in tobacco leaves. The fluorescence of BrERF72-GFP was predominately observed in the nucleus of epidermal cells, but GFP signal of positive control was found both in cytosol and nucleus (Fig. 4b). To examine the transcriptional activity of BrERF72, a BrERF72 fusing with the GAL4 DNA-binding domain-coding sequence (GAL4BD) construct was prepared, and introduced into yeast cells. Like the positive control (p53 + T-antigen), yeast cells expressing the BrERF72 grew well on SD plates without tryptophan, histidine, and adenine, and showed α-galactosidase activity (Fig. 4c), indicating that BrERF72 possesses trans-activation ability in yeast cells. Using the dual-luciferase reporter system, trans-activation of BrERF72 was further verified in tobacco leaves, which showed that expression of BrERF72 with the dual-luciferase reporter, as well as the transcriptional activator control VP16, remarkably elevated the LUC/REN ratio (Fig. 4d). Collectively, these data demonstrate that BrERF72 is a MeJA-induced transcriptional activator.

### Transient overexpression of *BrERF72* in tobacco leaves leads to accelerated leaf senescence

**Fig. 5:**
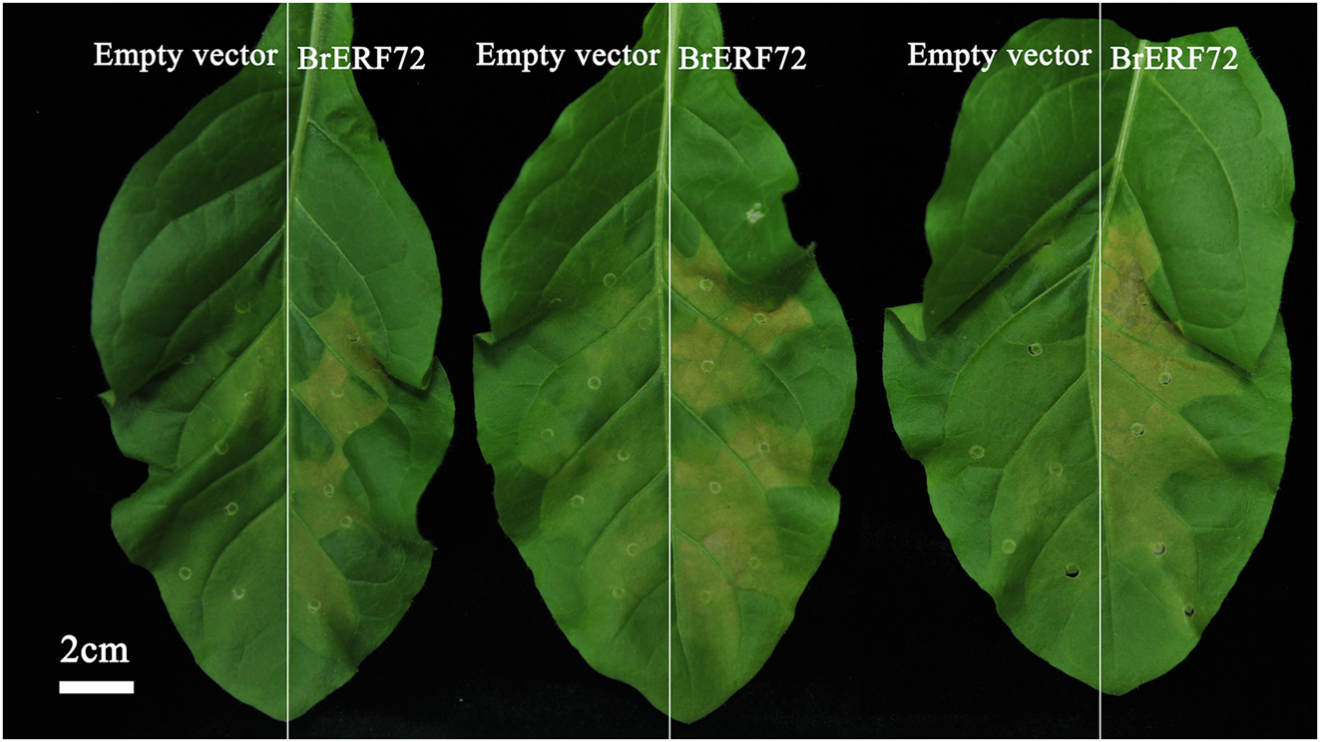
Transient overexpression of *BrERF72* promotes leaf senescence in *Nicotiana tabacum*. Appearance of *Nicotiana tabacum* leaves transiently expressed with empty vector (left side of leaf) and *BrERF72* (right). Three independent leaves were imaged at 5 days after infiltration

The possible association of BrERF72 with leaf senescence was further verified through the transient overexpression in tobacco leaves. As shown in Fig. 5, transient overexpression of *BrERF72* led to acceleration of leaf senescence, since more yellow lesions (the right blade of the leaves) were appeared, compared with the empty vector (the left blade of the leaves) (Fig. 5).

### BrERF72 directly targets *BrLOX4*, *BrAOC3*, and *BrOPR3* promoters through GCC or DRE/CRT elements

**Fig. 6:**
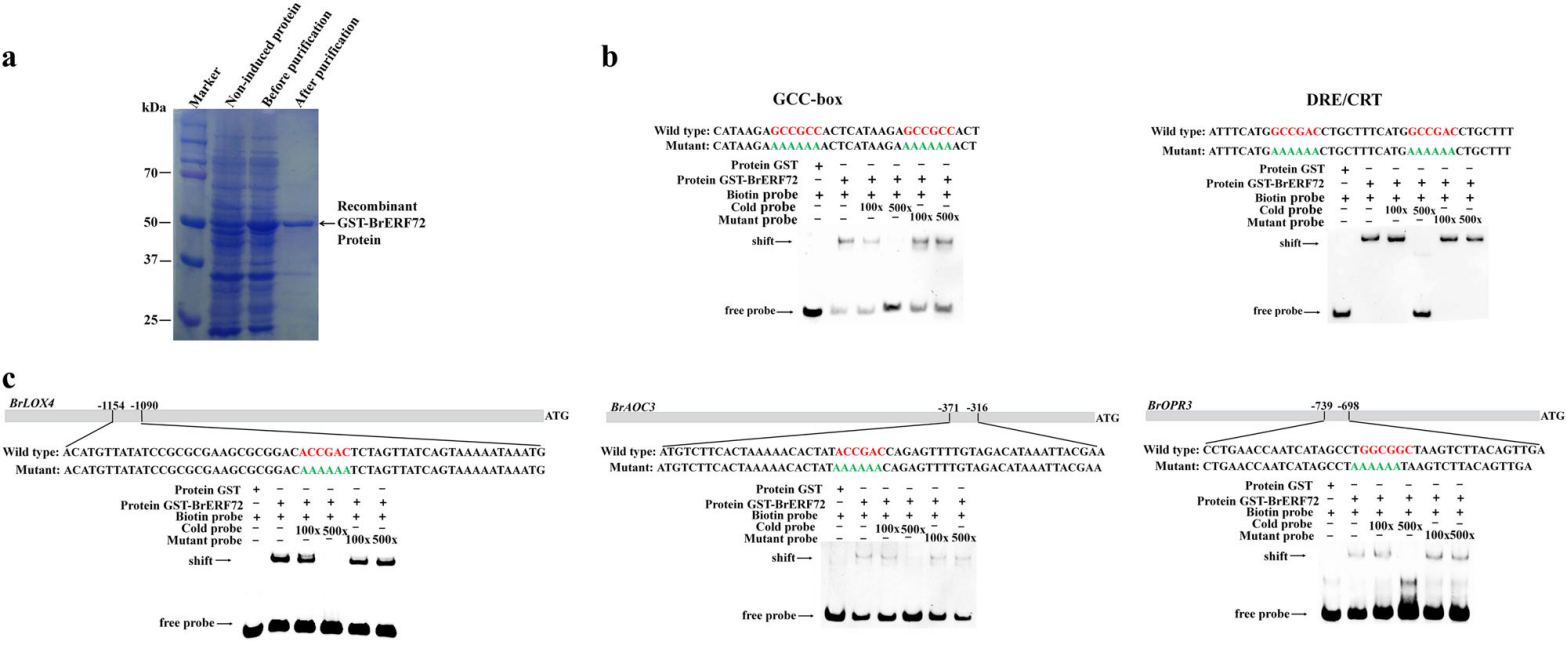
BrERF72 binds directly to *BrLOX4*, *BrAOC3*, and *BrOPR3* promoters containing GCC-box or DRE/CRT element by electrophoretic mobility shift assay (EMSA). **a** SDS-PAGE gel stained with Coomassie brilliant blue demonstrating affinity purification of the recombinant GST-BrERF72 protein used for the EMSA. **b** BrERF72 binds directly to GCC-box and DRE/CRT element. **c** BrERF72 binds to the GCC-box or DRE/CRT element of the *BrLOX4*, *BrAOC3*, and *BrOPR3* promoters. In **b** and **c**, the sequences of the wild-type probes containing the GCC-box or DRE/CRT element (red letters) were biotin labeled. The probes were mixed with the purified GST or recombinant GST-BrERF72 protein, and the protein–DNA complexes were separated on native polyacrylamide gels. Competition assays for the labeled probes were performed by adding an excess of unlabeled cold or mutated probes. Arrows indicate the position of shifted bands

Based on these above results, the regulatory mechanism of BrERF72 associated with MeJA-induced leaf senescence, especially whether BrERF72 can directly target JA biosynthetic genes, attracts our attention. Previously, it was shown that ERF TFs specifically bind directly to both GCC-box (AGCCGCC) and DRE/CRT [(A/G)CCGAC] motifs^18,45,46^. EMSA using the purified recombinant BrERF72 protein (Fig. 6a) was performed to investigate whether BrERF72 could recognize GCC-box and DRE/CRT motifs. As shown in Fig. 6b, the recombinant BrERF72 protein could directly bind to GCC-box and DRE/CRT motifs. Intriguingly, DRE/CRT motif was found in *BrLOX4* and *BrAOC3* promoters, and GCC-box motif was observed in *BrOPR3* promoters (Supplementary Text S1). Consistently, it was shown that the recombinant BrERF72 protein could bind directly to the DRE/CRT motif-containing fragment of the *BrLOX4* and *BrAOC3* promoters, and the GCC-box–containing fragment of the *BrOPR3* promoter, respectively (Fig. 6c). Moreover, increasing amounts of unlabeled wild probe obviously reduced BrERF72 binding to the biotin-labeled probes, whereas unlabeled mutant probes were failed to compete for BrERF72 binding (Fig. 6c), further indicating that BrERF72 directly targets the *BrLOX4, BrAOC3*, or *BrOPR3* promoters via the DRE/CRT or GCC-box motifs.

### BrERF72 activates the expression of *BrLOX4*, *BrAOC3*, and *BrOPR3*

**Fig. 7:**
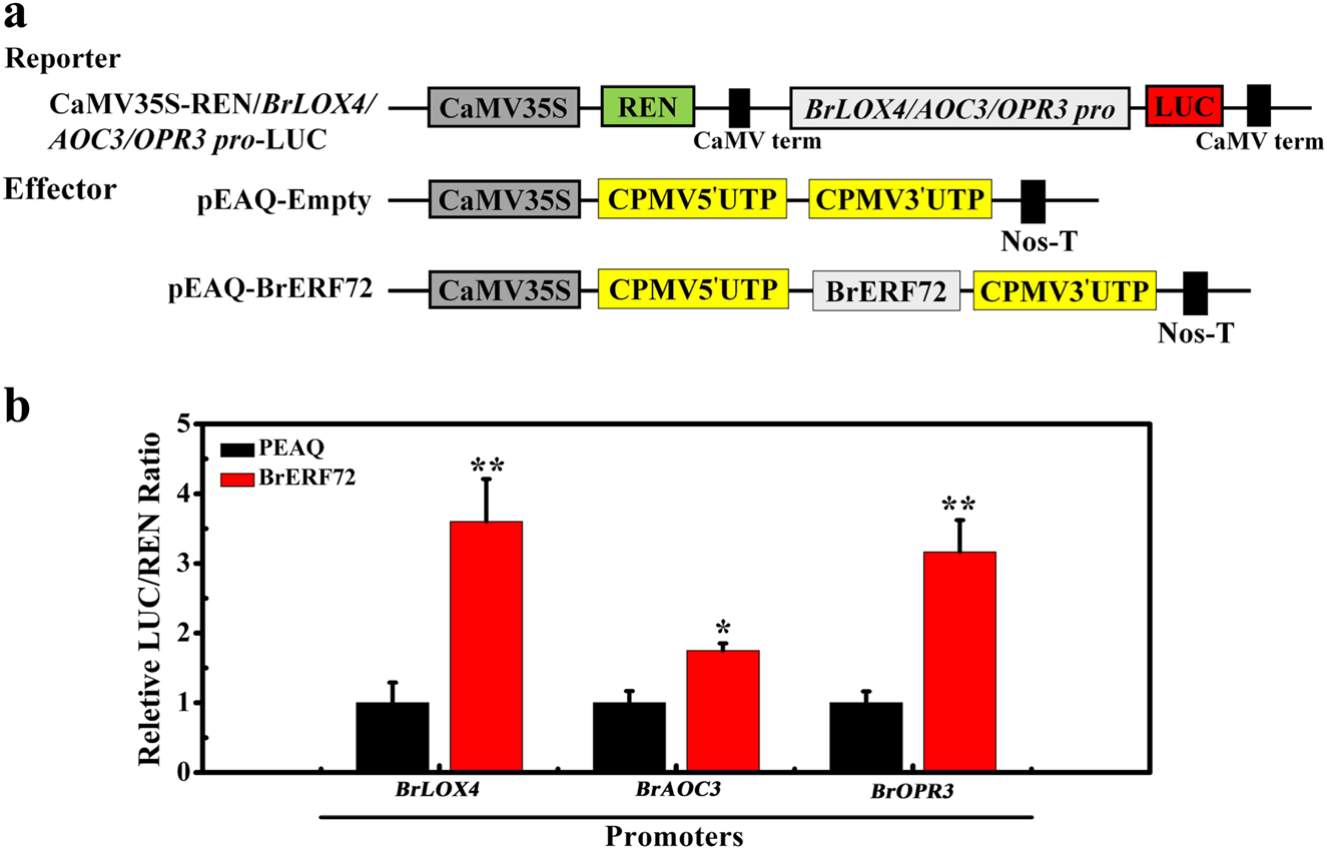
Dual-luciferase transient expression assay in tobacco leaves showing that BrERF72 activates the expression of *BrLOX4*, *BrAOC3*, and *BrOPR3*. **a** Diagrams of the reporter and effector vectors. **b** BrERF72 activates *BrLOX4*, *BrAOC3*, and *BrOPR3* promoters. Each value represents the mean ± SE of six biological replicates. Asterisks indicate significantly different values (Student’s *t* test: *P < 0.05 and **P < 0.01)

To detect the effect of BrERF72 on the expression of *BrLOX4, BrAOC3*, and *BrOPR3* after binding to their promoters, we performed dual-luciferase assays in tobacco leaves. *BrLOX4, BrAOC3*, or *BrOPR3* promoter was inserted into the pGreenII 0800-LUC plasmid and co-infiltrated into tobacco leaves with BrERF72 (Fig. 7a). As illustrated in Fig. 6b, when BrERF72 was co-transformed with *BrLOX4, BrAOC3*, or *BrOPR3* pro-LUC reporter, the value of LUC/REN ratio was remarkably increased, compared with the empty control. These data clearly indicate that BrERF72 activates *BrLOX4, BrAOC3*, and *BrOPR3* expression by directly targeting their promoters.

## Discussion

The phytohormone JA has been reported to promote leaf senescence in several species^10,19^. Our study also showed that exogenous MeJA treatment promoted leaf senescence of Chinese flowering cabbage, as MeJA treatment caused more reduction in Fv/Fm, ETR, and total chlorophyll content, while enhancing the expression of *BrSAG19*, and *BrPAO1*, *BrNYC1*, *BrPPH1*, and *BrSGR1*, resulting in more serious yellowing in MeJA-treated cabbage leaves (Fig. 1).

Several large TF families, including WRKY, NAC, and bHLH, are implicated in regulating JA-induced leaf senescence^13,14,19,47^. As one of the largest plant-specific TF families, AP2/ERF TFs differentially regulate diverse aspects of JA-mediated physiological processes^22,34^, for example, *Catharanthus roseus* ORCA3 is a MeJA-responsive AP2/ERF TF, and involved in JAs-stimulating monoterpenoid indole alkaloid biosynthesis^48^. Two AP2/ERF TFs AaERF1 and AaERF2 of *Artemisia annua* act as positive regulators of JAs-induced artemisinin biosynthesis^49^. AP2/ERF TF members also positively or negatively initiate the onset of leaf senescence in *Arabidopsis*, rice and tomato^29^. While their involvement in JA-promoted leaf senescence has been unclear. In this study, we identified a MeJA-inducible B-2 subgroup of ERF TF BrERF72 in Chinese flowering cabbage, and provided evidence showing that BrERF72 may be associated with MeJA-promoting leaf senescence through the regulation of transcription of JA biosynthetic genes *BrLOX4*,* BrAOC3*, and *BrOPR3*.

Accumulating evidence has supported the necessity of intact JA signaling for JA-accelerated leaf senescence^10,11,13,14,19,50,51^. JAZ (JASMONATE ZIM-DOMAIN) proteins and bHLH TFs MYCs are two key components of JA signaling, and their pivotal roles in JA-promoted leaf senescence have been extensively studied^10,50^. During senescence, endogenous JA content and expression of JA biosynthetic genes are strongly enhanced in senescent leaves and by MeJA treatment^11^. Similar results were also obtained in our study (Fig. 2). Interestingly, *Arabidopsis* class II CINCINNATA (CIN)/TCP TFs such as TCP4 positively influence JA biosynthesis through directly activating the transcription of *LOX2*^27^. miR319a/JAGGED and WAVY (jaw) can target class II TCPs^52^, and consequently *LOX2* expression and JA level in leaves of *jaw*-D plants are low, which leads to attenuate JA-induced senescence, while exogenous MeJA application rescues the senescence phenotype of *jaw*-D plants^27^. Recently, the class I TCP members TCP9 and TCP20 are shown to repress *LOX2* expression, and the *tcp9tcp20* double mutants exhibit an earlier initiation of leaf senescence^28^. In this work, we found that the Chinese flowering cabbage AP2/ERF TF BrERF72 was induced by MeJA, localized in the nucleus and possessed transcriptional activation ability (Fig. 4). Transient overexpression of BrERF72 in tobacco leaves also resulted in accelerated leaf senescence (Fig. 5). More importantly, BrERF72 activated the transcription of three JA biosynthetic genes *BrLOX4, BrAOC3*, and *BrOPR3* by directly targeting their promoters (Figs. 6 and 7). Our results, together with previous reports, reveal that besides intact JA signaling, transcriptional regulation of JA biosynthesis is also important for JA-modulated leaf senescence. However, it should be pointed out that transgenetic research such as stable overexpression of BrERF72 in *Arabidopsis* will be helpful to fully elucidate the involvement of BrERF72 in JA-promoted leaf senescence of Chinese flowering cabbage. Intriguingly, recently it is reported that a JA-induced TF MdMYC2 functions in JA-promoting ethylene biosynthesis via directly targeting MdERFs and ethylene biosynthetic genes *MdACS1* and *MdACO1* during apple fruit ripening^53^. Together with our work, these findings demonstrate that TFs, such as ERF and MYC2, are involved in crosstalk between JA and ethylene in mediating biological process.

Leaf yellowing, causing by chlorophyll degradation controlled by *CCGs*, is the most distinguishing feature of senescence. Recently, it was showed that the citrus CitERF13 directly targets *CitPPH* promoter and activates its expression, thereby leading to the acceleration of chlorophyll degradation and fruit degreening^42^. Three bHLH TFs of Arabidopsis MYC2/3/4, and three NAC TFs ANAC019/055/072, are involved in JA-promoted degreening via the direct mediation of a set of *CCGs* (*NYC1* and *SGRs*)^19^. Furthermore, MYC2 interacts with ANAC019 and they act synergistically to activate *SGR1* expression^19^. Leaf senescence is believed to be a complicated biological process concerning of many transcriptional regulators^54^. Therefore, identifying other targets such as *CCGs* of BrERF72, and dissecting other JA-responsive TFs such as MYCs, NACs, and WRKYs, as well as their connections in association with JA-mediated leaf senescence of Chinese flowering cabbage, will be our intriguing research topics in the future, which might extend our knowledge of the transcriptional regulation of leaf senescence in Chinese flowering cabbage.

## Conclusions

Taken together, we demonstrated that application of MeJA promoted leaf senescence of Chinese flowering cabbage. Furthermore, we characterized a MeJA-inducible transcriptional activator BrERF72, and showed that BrERF72 activated three JA biosynthetic gene expressions via directly targeting their promoters. We propose that BrERF72 might be associated with MeJA-prompted leaf senescence of Chinese flowering cabbage through directly regulating JA biosynthetic genes. Our findings provide novel cues about the transcriptional regulation of JA biosynthesis involving ERF TFs associated with JA-mediated leaf senescence.

## Electronic supplementary material

Supplementary data(PDF 63 kb)
