# Supplementary material for: Association of BrERF72 with methyl jasmonate-induced leaf senescence of Chinese flowering cabbage through activating JA biosynthesis-related genes
Source: Hortic Res. 2018 May 1;5:22. doi: 10.1038/s41438-018-0028-z (PMC5928098; doi:10.1038/s41438-018-0028-z)
Supplement: Supplementary file 1 — Supplementary data(PDF 63 kb) [file 41438_2018_28_MOESM1_ESM.pdf]

Table S1. Summary of primers used in this study.

Assay	Primer sequence			Restriction Site
RT-qPCR		Forward primer(5'-3')	Reverse primer(5'-3')	
	BrActin1	CGCTTAACCCGAAAGCTAAC	TACGCCCACTAGCGTAAAG	
	BrOPR3	CGGACCAGACCGTGTTGGCATTA	TCATAGGCACAAGCAAGTGAGGACA	
	BrAOC3	CTAACGGTCCAGATTTCTCCTCC	GGGCTGTTTCTATCTCCTTCGTT	
	BrLOX4	ATCAACAGAACTAGCCCGTCCG	GCACATACATCGGCAATGGTTTC	
	BrERF72	GAACTTGATGCTTCTGCCTGCGATG	TCCTCTCCTTCCGTTTCTCCGTC	
	BrSAG12	CACTGGCGGCTTAACCACTGAA	GAAGATTGGCTGTATCCTACGGC	
	BrSAG19	GCAAGCGAGCGTTGGTAAAGGT	GGGTTGATTCTTCCACTCCCTTC	
	BrPAO1	CAGCTTCAGCGACACTCACC	TCGCCGTGCTCTTCTTCGAT	
	BrPPH1	TATCTGATGCGCGGGTGGAT	TTCCCGACCAATGCTGGACT	
	BrNYC1	CGGAGACGGTGGCTAGAACG	TGCTTCTCCTGAGCCACGAC	
	BrSGR1	GTTGGGGTCCGCTTTGGGAA	AATCGAGCTAACCTGCGGGA	
Subcellular localization	*BrERF72-GFP-For*	ttctgcccaaattcgcgATGTGTGGCGGTGCTGTAATTTC		*AgeI*
	BrERF72-GFP-Rev	tagtcataccggtcgcTCCATACGACGCGATGACATCA		*AgeI*
EMSA	*pGEX-4T-1-BrERF72-For*	ggttccgcgtggatccATGTGTGGCGGTGCTGTAATTTC		*BamHI*
	pGEX-4T-1-BrERF72-Rev	agtcacgatgcggccgcTTATCCATACGACGCGATGACATCA		*NotI*
	EMSA -BrLOX4pro-For	ACATGTTATATCCGCGCGAAGCGCGGACACCGACTCTAGTTATCAGTAAAAATAAATG		
	EMSA -BrLOX4pro-Rev	CATTTATTTTTACTGATAACTAGAGTCGGTGTCCGCGCTTCGCGCGGATATAACATGT		
	EMSA -mLOX4pro-For	ACATGTTATATCCGCGCGAAGCGCGGACAAAAAATCTAGTTATCAGTAAAAATAAATG		
	EMSA -mLOX4pro-Rev	CATTTATTTTTACTGATAACTAGATTTTTTGTCCGCGCTTCGCGCGGATATAACATGT		
	EMSA -BrAOC3pro-For	ATGTCTTCACTAAAAACACTATACCGACCAGAGTTTTGTAGACATAAATTACGAA		
	EMSA -BrAOC3pro-Rev	TTCGTAATTTATGTCTACAAAACTCTGGTCGGTATAGTGTTTTTAGTGAAGACAT		
	EMSA -mBrAOC3pro-For	ATGTCTTCACTAAAAACACTATAAAAAACAGAGTTTTGTAGACATAAATTACGAA		
	EMSA -mBrAOC3pro-Rev	TTCGTAATTTATGTCTACAAAACTCTGTTTTTTATAGTGTTTTTAGTGAAGACAT		
	EMSA -BrOPR3pro-For	CCTGAACCAATCATAGCCTGGCGGCTAAGTCTTACAGTTGA		
	EMSA -BrOPR3pro-Rev	TCAACTGTAAGACTTAGCCGCCAGGCTATGATTGGTTCAGG		
	EMSA –mBrOPR3pro-For	CCTGAACCAATCATAGCCTAAAAAATAAGTCTTACAGTTGA		
	EMSA –mBrOPR3pro-Rev	TCAACTGTAAGACTTATTTTTTAGGCTATGATTGGTTCAGG		
Dual LUC assay	*pBD-BrERF72-For*	tcgccgaccggtaggcctATGTGTGGCGGTGCTGTAATTTC		*Stu I*
	pBD-BrERF72-Rev	aaccagagttaaaggcctTCCATACGACGCGATGACATCA		*Stu I*
	pEAQ-BrERF72-For	caaattcgcgaccggtATGTGTGGCGGTGCTGTAATTTC		*AgeI*
	pEAQ -BrERF72-Rev	agttaaaggcctcgagTTATCCATACGACGCGATGACATCA		*XhoI*
	0800-BrOPR3pro-For	tatagggcgaattggTGGGCTTGTATGGTTTGGGCC		*KpnI*
	0800-BrOPR3pro-Rev	ttggcgtcttccatggGAATTTCTGTGATTCTTTTTCTCTCA		*NcoI*
	0800-BrLOX4pro-For	tatagggcgaattggTACCTAGCTGGACTTATTGTTGGCGAC		*KpnI*
	0800-BrLOX4pro-Rev	ttggcgtcttccatggTCCACAAATGAATAAACTGTTGCAA		*NcoI*
	0800-BrAOC3pro-For	tatagggcgaattggAGGCAGTCAAACAATGTGTTGTTCA		*KpnI*
	0800-BrAOC3pro-Rev	ttggcgtcttccatggTTTATTTCGTATGTTAAGGGAAGAC		*NcoI*
Transcription activation	*pGBKT7-BrERF72-For*	catggaggccgaattcATGTGTGGCGGTGCTGTAATTTC		*EcoR 1*
	pGBKT7-BrERF72-Rev	gccgctgcaggtcgacgTTATCCATACGACGCGATGACATCA		*Sal1*

Text S1. Promoter nucleotide sequences of *BrOPR3*, *BrLOX4 and BrAOC3*. GCC-box GCCGCC or DRE elenment A/GCCGAC is marked by red and boxed. Translation start site (ATG) was shown in yellow box.

BrLOX4 (NW_008711256.1) promoter

TCGGCTGGAAGCTAAATCGGCTGGCAGCTTTTGGTCAAACAACCTCTGAAGGAAAAGCAGACGAAGCCACATTAA
AAGTCCTTATAGAAATGATGGATGCCAACAATTGCCTTGCAAAGGTTTTTAGACGGGTTCGAGAACGCTATGAGGC
AAACTCTGAACAAGATTTTACAGTCTCATATCTGATAAAGGGAATGGCAAACAATATGACTTACCTCAATCAAATGA
GGTAGCTGGACTTATTGTTGGCGACATGTCAGACACTATCTGTGAGAGAGATATAGTCGTCCAGTTTCAGTCAACTA
ATTTGAAGGAGATACGAGATGATCATCCGCTATACATGAACCTCCAGTTCCCACTTTTATTCTCTTATGGTGATTATG
GTTTCAATACTGAAATCCTTTTGCATTTAGAGGAAGGAAGCTCGAGGACAAGAAAATTTCTCAGTATCCGATAATTT
TATGCTTCCCAAATACAAACGCGGTTAAAAAAAAGAATAACACTCATTAAATCTGGACAATTTTGTTACTTAATTAA
ACATGTTATATCCGCGCGAAGCGCGGACACCGACTCTAGTTATCAGTAAAAATAAATGAAAAGTTTAAGCTGAACA
TGTCCGTATTATTCAGAAAATCTAAGACTTGTTTTTTAAAAATCAGCGTACTTAGGTAAACTATGAATATTCCAATTA
AAAAAACGTGTGGACTGAGAATAAGCTTTCTCTCTCCCTAAGCTAAAGCATATAAATAGCTGTCGCCTGCACATTAT
TTGTAATCAGAACGCACTAACAAAAGCTCTCAACTTCTTCTTGAAAGCTTTAACCTTTTTCTGCAATCTCTTATCAC
TTCCAAGAAACAAAAACCCTCAGATTCAATCATTAGTTTGTACTAATTAAATTCATTTGTGGAATG

BrAOC3 (NW_008711386.1) promoter

AGGCAGTCAAACAATGTGTTGTTCAAATCCGTTCTATCAGATCTGAACATTTTCATAAATATTGTTTGGAGAAAAAA
TTATGTGGGTTAACTACTTTCCTTCGAAATTCGATTCTATCAAAATAATTTACACTAATAATGATGTCTCATCATTGTG
AATTAGAACCTCAACATTTCATTTATAACGATGTTTCAACAAAAATGATACTTATATTTCGTAGTAGCAGAAATTAAA
ATGTTTAGAATATTTCCAATAGTTCATTCTATTTTTTTAACGTAGAATAATTGTATAGTAGAGTTAAATTTTACTTTGAT
GATATTCTACTTTAAAATAAAAATAGAATGATGAATAAAAAAATAATGAATTATTTTATTTATAGAATAAACTTAGTTT
TTACTATATATCGAGTGAAAAATAGAATATCATTAAAACATTTTTACTTTAAATTATATTTTAGAAGAAAAAAATATAT
TGAAATTAGAAATGCTCTTATAGCTACGTCTTGTTCGACTATACCAACTAGTCACAAAGAAATATTTTACATCGCTGA
AATATTAAAAGTTTTGTGACATACGACTGATCACTATGACTACTTGTAGAAACATGCCAATGAACAATATTTGGTTAT
TAGTTGCAATGAACAAAAAATTAACACAACTGAACAAAGTTACTAATGTCTTCACTAAAAACACTATACCGACCAG
AGTTTTGTAGACATAAATTACGAAACGTATATCTTTTGCCTGTTTTGGTATCTTTCCATTACTTGGAGATCTCGTACTC
TACTCTCTACGTAATGACTCGTTTTCAGTCAATTTCTCAAGCTTGCGCTTTGAAACCGTCCATGGGAAACCGAGTCG
AAATTTCACCAAAGCCATCTTTCATTTAGAAACCGGCCACTTTCTAAATATACGCACGTGCAAGTTACATCATCCCC
GCATCCGTAGACTAAACACACTCATTATATAAACAAGATCCTCCCAATCTCTACTCTCATAATCAATCAAACCAAGTT
CCAAGTCTTCCCTTAACATACGAAATAAAATG

BrOPR3 (NW_008711256.1) promoter

TGGGCTTGTATGGTTTGGGCCGCGGGCCGTTTATAGCATTTAGACTGTAGCCGGTTTGGCTTATGGGCTTCGTGCCT
TTTTGTTTATGCGTTTGGGCTTCAGACTATGTAATTAGGCTTGGCCCGGTATTATTATTAATCTAAATAAAATCTTGAC
GGAAAAAAAAAAAAAAAGAATGTCACACATGTACAATATGTACAAAGGATCTTTTTTGAATAAGCAAGAAACAGC
TAGACAGATACATACCTCCGGTCCCACCTGAACCAATCATAGCCTGGCGGCTAAGTCTTACAGTTGAATTGTTCATG
GGTTGTCCATATGGTGATGTGAACCATACACATTACACTAAATACTGATAAAAAAGGATACCAACTCTTCTATTCGA
CTTATGTTTTTCGGTCTTGAATTTCTAAATCAGTTACTCTAAATCAACAACTTATGTTTTTCAAATCTGAACTTTTATG
TTACTCTAAATCTTCTATTCGAGCATTCATTTCAGTCTCAAATCCCGTTATGTATGATGCTATCTTTCTTTCCTTTTTAC
TCGAACACCTTAACCTTTTGTTCTGTCGGTGGTCAAAGTTGGACAAAGTCAATCCAATTATTTTCTTTTTAATTATTA
AAATTATATGAGGCCCATTAAAGTTTTGTTCTGTAATTATTCTCTAAAAGTCGAATCTCAACTGTAGGACGGAGTAA
GAAAGAAACGAGCATTATTGTGGTTAAGTTCAAAGTGGTGGTTACTCTCTAATGTCGTCAATGCATACGTCATGTAA
ATGCAAGTGGATGGGGTCCACGGATGCATGCGTCCTCAATGACGTCCACGTCCAAAGAAAAAACTTACTTTCCTAA
TATGTATATCTAACCAAATGTGTATATTTGAACAAAAAAAAAAAAACCAAATGTGTATATATATATGTCGCAGATATAAC
CATCTTCGTCCACAACTCACCAAAAACTTGAAAGTGTTTTGAGAGAAAAAGAATCACAGAAATTCATG
